# Transcriptomic Analysis Reveals Important Roles of Lignin and Flavonoid Biosynthetic Pathways in Rice Thermotolerance During Reproductive Stage

**DOI:** 10.3389/fgene.2020.562937

**Published:** 2020-09-15

**Authors:** Zhenzhen Cai, Fengyu He, Xin Feng, Tong Liang, Hongwei Wang, Shuangcheng Ding, Xiaohai Tian

**Affiliations:** ^1^Engineering Research Center of Ecology and Agricultural Use of Wetland, Ministry of Education, Agricultural College, Yangtze University, Jingzhou, China; ^2^Hubei Key Laboratory of Waterlogging Disaster and Agricultural Use of Wetland, Agricultural College, Yangtze University, Jingzhou, China; ^3^Hubei Collaborative Innovation Center for Grain Industry, Agricultural College, Yangtze University, Jingzhou, China

**Keywords:** rice, heat stress, transcriptome, spikelet, meiosis, lignin and flavonoid biosynthetic pathways

## Abstract

Rice is one of the major staple cereals in the world, but heat stress is increasingly threatening its yield. Analyzing the thermotolerance mechanism from new thermotolerant germplasms is very important for rice improvement. Here, physiological and transcriptome analyses were used to characterize the difference between two germplasms, heat-sensitive MH101 and heat-tolerant SDWG005. Two genotypes exhibited diverse heat responses in pollen viability, pollination characteristics, and antioxidant enzymatic activity in leaves and spikelets. Through cluster analysis, the global transcriptomic changes indicated that the ability of SDWG005 to maintain a steady-state balance of metabolic processes played an important role in thermotolerance. After analyses of gene ontology (GO) and Kyoto Encyclopedia of Genes and Genomes (KEGG) pathway enrichment, we found that the thermotolerance mechanism in SDWG00 was associated with reprogramming the cellular activities, such as response to abiotic stress and metabolic reorganization. In contrast, the down-regulated genes in MH101 that appeared to be involved in DNA replication and DNA repair proofreading, could cause serious injury to reproductive development when exposed to high temperature during meiosis. Furthermore, we identified 77 and 11 differentially expressed genes (DEGs) involved in lignin and flavonoids biosynthetic pathways, respectively. Moreover, we found that more lignin deposition and flavonoids accumulation happened in SDWG005 than in MH101 under heat stress. The results indicated that lignin and flavonoid biosynthetic pathways might play important roles in rice heat resistance during meiosis.

## Introduction

With an increase in the frequency and intensity of extreme heat waves, high-temperature stress has become one of the severe limiting factors affecting crop production and distribution worldwide (Lobell and Gourdji, 2012; Wang et al., 2016; Yang et al., 2017). The mean global temperature has been predicted to rise 2.5–5.8°C by the end of the 21st century (IPCC 2014). The yield of crops significantly negatively correlated with temperature rise at least in the three major crops, rice, maize, and wheat (Yu et al., 2012; Kawahara et al., 2013; Li et al., 2016). The data showed that rice yields would decline by 10% with each 1°C increase in average minimum temperature (Peng et al., 2004).

The reproductive stage of rice was the most sensitive phase to heat stress. Of all the reproductive processes, the most susceptible stages to heat stress were flowering and microspore meiosis (Satake and Yoshida, 1978; Jagadish et al., 2007). Jagadish et al. (2007) reported that cumulative temperature over 33.7°C even as short as 1 hour at anthesis could lead to infertility in *indica* rice. Tian et al. (2009) observed that a mean temperature of 30°C, with approximately 70% relative humidity and low wind speeds, during the flowering period for longer than three days could lead to significantly lower seed setting in rice plants (Tian et al., 2009). Several previous studies have also revealed that pollen abortion and floret sterility were induced by exposure to a high temperature of about 33°C at meiosis of microspore mother cell stage (Prasad et al., 2006; Krishnan et al., 2011). In comparison to flowering, rice pollen abortion and floret sterility were relatively less susceptible to high temperature at the meiosis stage (Matsui et al., 2000; Jagadish et al., 2010, 2014; Shah et al., 2011). During meiosis stage, male sterility induced by high temperature appears to be associated with the poor pollen development (Matsui et al., 2000; Ku et al., 2003). Overall, the spikelet fertility was primarily determined by successful pollination and fertilization, thus the heat-induced decrease in spikelet fertility at reproductive stages was mainly attributed to lack of full development of pollens, abnormal anther dehiscence, poor pollen viability, and failed germination and/or elongation on the stigma (Satake and Yoshida, 1978; Matsui et al., 2000; Jagadish et al., 2010; Yu et al., 2016). Although many physiological studies have contributed to our understanding of heat-induced pollen fertility injury, molecular analyses are still largely lacking.

In recent decades, some heat stress response pathways and key regulators that function during anther development have been clarified (Frank et al., 2009; Jagadish et al., 2010). Several studies showed that during heat stress the imbalance between ROS and ROS scavenge system existed, and then induced the initiation of tapetum programmed cell death (PCD) (Suzuki et al., 2001; Yu et al., 2016). The genes participating in starch and sucrose metabolism in anthers were reported as crucial metabolic and transcriptional components in the acquisition of thermotolerance (Scharte et al., 2009; Li et al., 2015). Additionally, some researchers have also reported that plant hormones were key regulators of pollen development and thermotolerance (Tang et al., 2007; Mittler et al., 2012; Bokszczanin et al., 2013). Thus, different signal transduction events were triggered to establish homeostasis of metabolic processes by altering the composition of certain transcripts, proteins, metabolites, and lipids (McClung and Davis, 2010). Furthermore, a lot of heat-responsive genes and proteins, including heat shock proteins, antioxidant enzymes, and various transcription factors, have been identified and their functions well elucidated (Guo et al., 2008; Ramamoorthy et al., 2008; Scafaro et al., 2010).

However, currently, our understanding remains limited on how plants respond to heat stress at the whole plant level, especially on the core pathways leading to increased thermotolerance in the crucial floral organs that determine reproductive success. Therefore, it is necessary to discover the genes regulating thermotolerance and investigate the intrinsic molecular mechanism in new thermotolerant germplasms. Here, physiological and transcriptome analyses were used to characterize the difference between two germplasms, heat-sensitive MH101 and heat-tolerant SDWG005. SDWG005, belonging to *Oryza sativa* spp. *indica*, is an African landrace that exhibits heat-tolerant, while MH101, a conventional *indica* variety rice, is very sensitive to high temperature. We firstly compared the different responses to heat stress between SDWG005 and MH101 by observing their phenotypic characteristics, including pollen viability, pollination properties, and antioxidant enzymatic activities. Then we analyzed the global transcriptomic changes in response to heat stress in the two genotypes, and identified the important roles of lignin and flavonoids biosynthetic pathways in rice thermotolerance during meiosis. The results will lay an important foundation for understanding thermotolerance mechanisms in rice spikelet, and provide insights into the underlying molecular basis of heat responses.

## Materials and Methods

### Plant Materials and Treatments

Two rice (*Oryza sativa* L.) genotypes, SDWG005 and MH101, were used in this study. SDWG005 belonging to *Oryza sativa* spp. *indica*, is an African landrace that exhibits heat-tolerant, while MH101, a conventional *indica* variety rice, is very sensitive to high temperature. Twenty-day-old seedlings were transplanted into a plastic pot (inner diameter 30 cm, height 30 cm) containing 12.5 kg soil and 8 g N: P: K compound fertilizer (26:10:15). The tillers were cut off leaving only the main stem during the plant culture. The plants were grown until the meiosis stage, when the distance between the pulvinus of the first leaf and second leaf from the top was about 2 cm. One day later, the rice plants were transferred to a growth chamber (AGC-MR, ZhejiangQiushi Environment Co., Ltd, Zhejiang, China) and started the heat treatment with a 14-h-day/10-h-night cycle and a 2-h change on the temperature simulating the local typical heat stress weather. The actual temperature regimes were 26, 30, and 34°C in daily mean temperature, respectively, and their corresponding daily maximum temperature were 28, 36 and 38°C. After heat treatment for three days, spikelet and flag leaves were collected from both heat stress and control plants (six independent plants of each) for each one of MH101 and SDWG005 for the extraction of total RNA or detection of antioxidant enzymatic activities. All collected samples were immediately frozen in liquid nitrogen and stored at −80°C. Before anthesis (after heat treatment for eight days), the rice plants were moved out from growth chamber, and analyzed for the pollen viability and pollination properties.

### Determination of Pollen Viability

The spikelets in the panicle containing anthers were collected on day 3 of the heat stress treatment. The pollen grains removed from the anthers were stained with 1% KI/I_2_ on a glass slide, and subsequently observed and photographed with a magnification of 100× under a light microscope (DM4000B; Leica, Wetzlar, Germany). The pollen grains were divided into fertile and sterile groups, based on their colors. In each photo, 30–150 pollen grains were analyzed (10 repetitions). Percentage of pollen viability = (the number of fertile pollen grains/the total number of pollen grains) × 100%.

### Determination of SOD, POD, APX, and MDA

Frozen spikelets or flag leaves (0.5 g) were ground into a fine powder in liquid nitrogen and then homogenized in a 50mM sodium phosphate buffer (pH 7.0). The homogenate was centrifuged at 13,000 g for 15min at 4°C, and the supernatant was stored in aliquots at −20°C until further use. The superoxide dismutase (SOD) activity was determined using the method of Giannopolitis and Ries (1977). The method of Maehly and Chance (1954), in which guaiacol is converted to tetraguaiacol and monitored at 470 nm, was used to determine the peroxidase (POD) activity. Ascorbate peroxidase (APX) activity was measured by the method of Bonnecarrère et al. (2011). The Malondialdehyde (MDA) content was measured by a modified TBA (Thiobarbituric acid) method as described (Peever and Higgins, 1989).

### Determination of Lignin, Flavonoids, and Phenolics Content

Frozen spikelets were collected, oven-dried at 65°C to constant weight, and mortar ground over an 80-mesh sieve. Lignin content was determined from the cell wall fraction (removing of other compounds by phosphate buffer, 1% Triton X-100, 1 M NaCl and acetone) by thioglycolic acid reaction (Kovacik et al., 2007). Total phenolics were extracted by 80% methanol and calculated from the calibration curve prepared using gallic acid by the Folin-Ciocalteu method (Kovacik et al., 2007). The aluminum chloride colorimetric assay was used to determine the flavonoid content of both free phenolics and bound phenolics of plant extracts, of which, the total flavonoid content was established using a calibration curve and expressed as mg/g FW (Djeridane et al., 2006).

### RNA Isolation and cDNA Library Construction

The spikelet sampled from control (28°C) and heat (36°C, 38°C) treated for MH101 and SDWG005 were collected for isolating RNA and cDNA library construction. Total RNA was prepared from spikelet with the Promega RNA extraction kit (Promega, China, LS1040). RNA integrity and quantity were measured with a Nanodrop N60 spectrophotometer (Implen) and an Agilent’s Bioanalyzer 2100. The triplications were prepared for each sample, resulting in a total of 18 libraries sequenced by the Illumina HiSeq X Ten system to produce 150 bp paired-end reads per library. Sequencing was performed at Beijing Genomics Institute (Wuhan, China).

### Detection of Differentially Expressed Genes

The rice genome version Rice Genome Annotation Project (RGAP7) was downloaded from http://rice.plantbiology.msu.edu (Kawahara et al., 2013). RNA-seq reads were aligned to the rice genome using the alignReads mode of STAR 2.4.1c (Dobin et al., 2013) with the default settings. FeatureCounts 1.4.6 (Liao et al., 2014) was used to summarize uniquely mapped paired-end reads and count fragments with “-t exon” and “-g gene_id”. For MH101 or SDWG005, differentially expressed genes (DEGs) were derived from the comparison between control (28°C) and heat treatment under different temperatures (36°C, 38°C). DEGs were detected using the edgeR package (version 3.24.3) (Robinson et al., 2010) with a false discovery rate (FDR) adjusted *P*-value cutoff at 0.05 and an absolute value of log_2_ transformed fold change relative to control (log_2_FC) cutoff at 1.

### Principal Component and Cluster Analyses

PCA was performed with the prcomp function of the base “stat” package (version 3.5.1) in R, using the log_2_ transformed raw reads counts as input. Differentially expressed genes in at least MH101 or SDWG005 were subjected to hierarchical clustering using the hclust function in R (distance: euclidean; method: ward.d) and the log_2_FC was used as the input. The hierarchical clustering was manually renumbered for simplicity of comparison.

### GO Categories and KEGG Pathways Analysis

To identify the biological functions over-represented within the DEGs between up- and down-related DEGs or each numbered cluster, functional enrichment analyses were performed using the AgriGO program (Du et al., 2010). Singular enrichment analysis was used to identify enriched GO terms with the FDR adjusted *P*-value cutoff of 0.05. The Kyoto Encyclopedia of Genes and Genomes (KEGG) pathways (Ogata et al., 1999) enriched within DEGs were analyzed with the Fisher’s exact test and the Benjamini-Hochberg multiple testing correction method.

### Real-Time PCR Analysis

The relative expression of the selected genes was measured using quantitative real time-PCR (RT-qPCR) in 96-well plates using the ABI7500 Real-Time PCR Systems (Applied Biosystems, United States). The PCR reaction system consists of 1 μg cDNA, 200 nM primers, and 5 μl SYBR Premix Ex Taq II (Takara, China), and the reaction volume is 10 μl. The PCR reaction was conducted with the following conditions: 10 min at 94°C, 40 cycles of 15 s at 94°C, and 30 s at 60°C. The expression level of the *O. sativa β-tubline* gene was used as internal control. For quantification, the 2^–ΔΔCq^ method was used, and variations (standard errors) were calculated from three biological replicates. Primers for qRT-PCR are listed in Supplementary Table S1.

## Results

### Pollen Viability and Pollination Properties of SDWG005 and MH101 Display Differences Under Heat Stress

**FIGURE 1:**
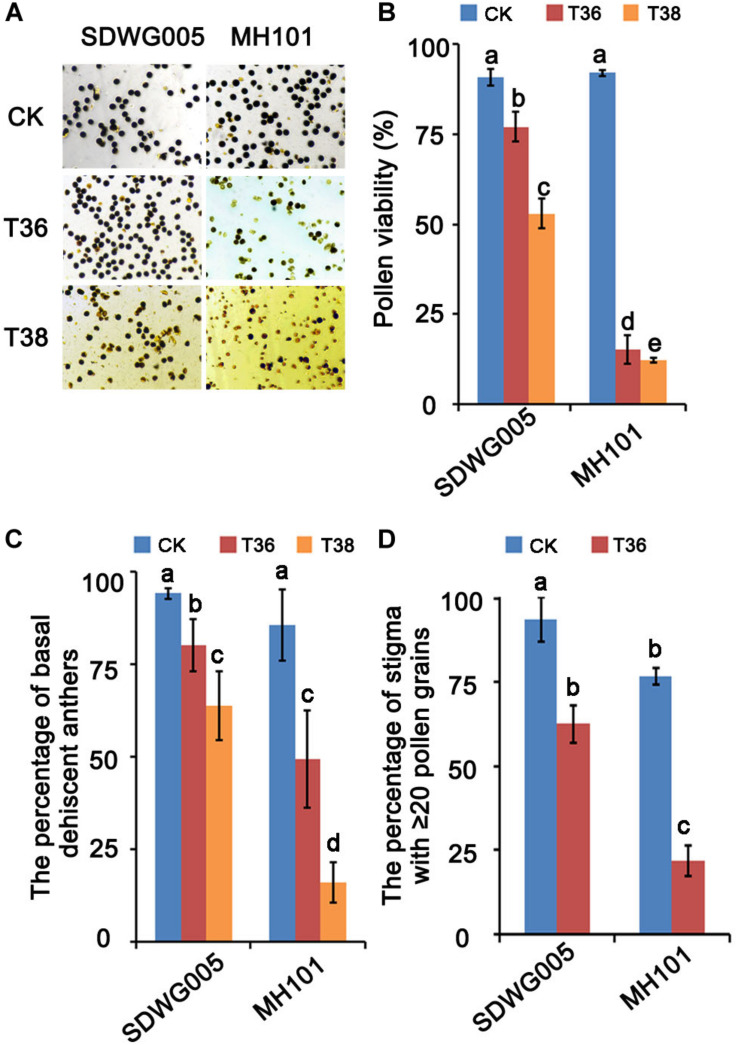
Effect of heat stress on pollen viability and pollination properties of SDWG005 and MH101. **(A,B)** Differences in pollen viability of SDWG005 (heat-tolerant) and MH101 (heat-susceptible) exposed eight days to different temperature treatments. **(C)** The percentage of basal dehiscent anthers under high-temperature treatments. **(D)** The percentage of stigma with ≥ 20 pollen grains at anthesis. CK, T36 and T38 indicate normal temperature (*T*_max_ = 28°C) and high temperatures T36 (*T*_max_ = 36°C) and T38 (*T*_max_ = 38°C). Each data point represents the mean ± SD (*n* = 3) of three biological replicates. Significant differences are calculated by One-way ANOVA with Duncan’s multiple test (SAS Institute, Inc., Cary, NC, United States). Different letters indicate statistical differences between samples at *P* = 0.05.

Previously, six rice varieties from various regions of Africa were treated with high temperature in flowering period to evaluate the thermotolerance based on the fertilization rate under different high-temperature treatments (Liu et al., 2019). The results showed that SDWG005, an extremely strong heat-resistant African landrace, was weakly affected by heat stress. In contrast, MH101 was more sensitive to heat stress. To further confirm their differential responses to high temperature, both varieties were treated under varied heat regimes for about eight days at meiosis stage, and the pollen viability and pollination properties were analyzed. The results showed that heat stress at the meiosis stage significantly affected pollen viability and pollination properties in rice plants, and the extent of the viability reduction and deficient pollination were dependent on the genotypes and temperature (Figure 1). Compared with the normal growth control (CK), treatments of T36 (T_max_ = 36°C) and T38 (T_max_ = 36°C) resulted in 76.83% and 79.73% reduction, respectively, in pollen viability in MH101 plants, whereas in only 13.74% and 37.82% reduction in SDWG005 plants (Figures 1A,B). The percentage of basal dehiscent anthers was measured in the flowers of SDWG005 and MH101 plants at anthesis, and the results showed that heat stress significantly reduced the rate of basal anther dehiscence in the two genotypes (Figure 1C). Compared to the normal growth control, treatments of T36 and T38 led to 13.98% and 30.41% decrease, respectively, in SDWG005. In MH101 plants, treatments of T36 and T38 caused 36.55% and 69.6% decrease, respectively, significantly greater than in SDWG005 (Figure 1C). We further compared the number of pollens on the stigma in SDWG005 and MH101 under different high-temperature treatments, and analyzed the percentage of stigma with ≥ 20 pollen grains. As shown in Figure 1C, MH101 was more significantly affected than SDWG005 by temperature with regard to the pollen number on stigma (Figure 1C). These results indicated that the pollen viability and pollination properties of SDWG005 decreased much slower than those of MH101 when temperature increased, confirming that SDWG005 (heat-tolerant) is much more tolerant to heat stress than MH101.

### SDWG005 Carries Higher SOD, POD, and APX Activities Than MH101, and Is Less Affected by Heat

**FIGURE 2:**
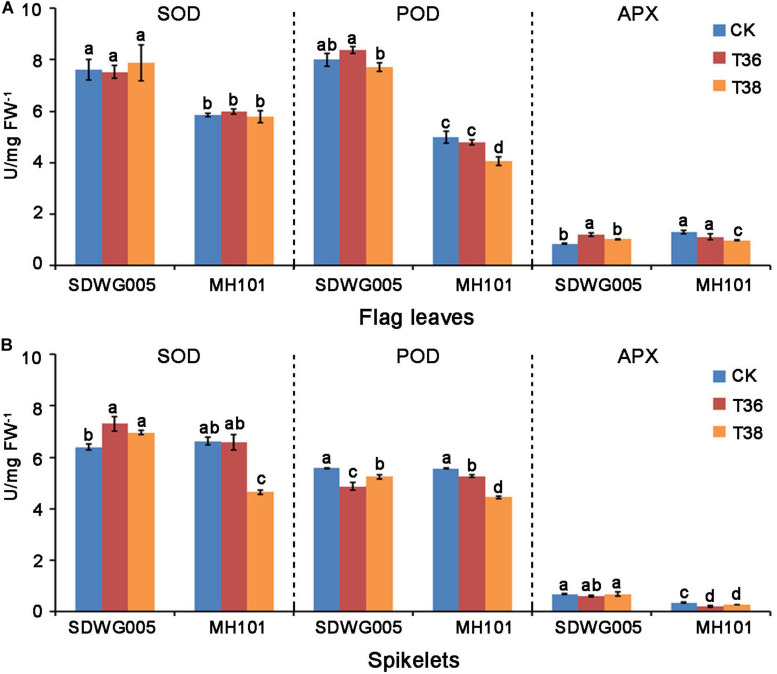
SOD, POD, and APX activities in rice flag leaves and spikelets under different high-temperature stresses. **(A)** SOD, POD, and APX activities in rice flag leaves when exposed three days to different temperature treatments. **(B)** SOD, POD, and APX activities in rice spikelets when exposed three days to different temperature treatments. CK, T36 and T38 indicate normal temperature (about 28°C) and high temperatures T36 (*T*_max_ = 36°C) and T38 (*T*_max_ = 38°C). Each data point represents the mean ± SD of three biological replicates. Significant differences were calculated by One-way ANOVA with Duncan’s multiple test (SAS Institute, Inc., Cary, NC, United States). Different letters indicate the statistical difference between samples at *P* = 0.05.

Enzymatic antioxidants, such as superoxide dismutase (SOD), peroxidase (POD), and ascorbate peroxidase (APX), are important reactive oxygen species (ROS) scavenging enzymes in reducing heat-induced ROS levels. We then examined the antioxidant enzymatic activities of SOD, POD, and APX in the flag leaves and spikelets of SDWG005 and MH101, respectively, after T36 and T38 treatments for three days. Whether upon heat stress or not, SDWG005 showed higher SOD and POD activities than MH101 in flag leaves (Figure 2A). No significant differences among SOD activity were observed in the flag leaves of two varieties under high-temperature stress. However, MH101 showed a significant decreases in POD and APX activities in flag leaves under T38 treatment; while SDWG005 was little affected, or even displayed a slight increase in POD and APX activities at T36 treatment (Figure 2A). Under normal conditions, SDWG005 and MH101 showed no differences among SOD and POD activities in spikelets, expect for APX activity (Figure 2B). Whereas MH101 had clear decreases in SOD, POD, and APX activities in spikelets under heat stress, SDWG005 only had a significant drop in POD activity at T36 (Figure 2B). These results suggested that SDWG005 carries higher SOD, POD, and APX activities than MH101 in flag leaves and spikelets, and is less affected by heat.

### Transcriptome Sequencing, Detection of DEGs and Validation Analysis by RT-qPCR

**FIGURE 3:**
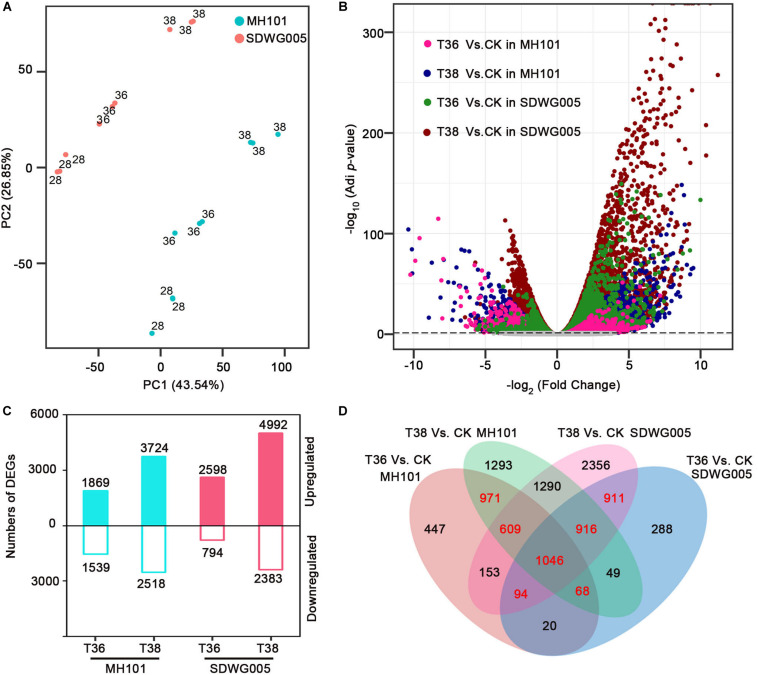
Analysis of DEGs in rice varieties in response to heat stress. **(A)** PCAs of log_2_ transformed raw read counts for CK, T36, and T38 for MH101 and SDWG005, respectively. **(B)** Volcano plot of expressed genes under different heat stress. Log_2_ transformed FC was plotted against the -log_10_ transformed adjusted *P*-value. Pink, blue, green and red dots represent DEGs of T36 relative to CK in MH101, T38 relative to CK in MH101, T36 relative to CK in SDWG005, T38 relative to CK in SDWG005, respectively. The gray line represents the –log_10_ transformed adjusted *P*-value cutoff. **(C)** Number of up- (log_2_FC ≥ 1 and FDR adjusted *P*-value ≤ 0.05) and down-regulated (log_2_FC ≤ -1 and FDR adjusted *P*-value ≤ 0.05) DEGs detected under different heat stress treatments relative to control for MH101 and SDWG005, respectively. **(D)** Venn’s diagram of the comparison of DEGs under different heat stress treatments in MH101 and SDWG005.

To characterize the differential transcriptomic responses to heat stress between SDWG005 and MH101 during meiosis, we subjected spikelets of SDWG005 and MH101 to 36°C (T36) and 38°C (T38) treatments for three days or grown at 28°C (T28, control). Thus, for each one of SDWG005 and MH101, there were three conditions, including two treatments (T36 and T38) and one control (T28). With three biological replicates for each condition, a total of 18 cDNA libraries were constructed, and sequenced using the Illumina deep sequencing platform. After filtering out the low-quality reads, a total of ∼369 million paired-end clean reads were obtained with an average of 86.34% that were uniquely mapped to the rice reference genome (RGAP7^1^) (Supplementary Table S2). Principal component analysis (PCA) was performed to capture the overall variance among treatments. Principal components 1 (PC1) and 2 (PC2) captured 43.54% and 26.85% of the variation in the gene expression data, respectively. A clustering effect based on temperatures was observed along the PC1 dimension, while the PC2 dimension separated the effect of materials (Figure 3A). Moreover, replicates of each one of the heat-stressed conditions for MH101 or SDWG005 were clustered nearby but were separated from the corresponding controls.

To identify DEGs for each treatment relative to the control of MH101 or SDWG005, edgeR (Robinson et al., 2010) was used and the outputs were further filtered using the criteria of the absolute value of log2 transformed fold change (| log_2_FC|) ≥ 1 and false discovery rate (FDR) adjusted *P*-value ≤ 0.05. As expected, numerous DEGs from the four treatments (T36-SDWG005, T38-SDWG005, T36-MH101, and T38-MH101) relative to the corresponding control were detected, with significantly higher/lower expression in SDWG005 (green and red) or MH101 (pink and blue) color-coded in Figure 3B. Compared to the control, a total of 3408 DEGs (1869 up- and 1539 down-regulated) in T36-MH101 and 6242 (3724 up- and 2518 down-regulated) in T38-MH101 were identified (Figure 3C); while a total of 3392 (2598 up- and 794 down-regulated) in T36-SDWG005 and 7375 (4992 up- and 2383 down-regulated) DEGs in T38-SDWG005 were identified. Additionally, as illustrated in the diagrams (Figure 3D), the partial overlap of DEGs among treatments were examined, and the results showed that in MH101(including 2694 DEGs) or SDWG005(including 2967 DEGs), a total of 4615 DEGs (in red) responded to T36 and T38 heat treatments (Supplementary Table S3).

To further examine the response to heat stress, DEGs with significant changes (log_2_FC values) in T36 treatment were plotted against those of T38 treatment for MH101 and SDWG005, respectively (Supplementary Figure S1). Consistent with the PCAs analysis, transcriptomic response to different heat stress treatments were highly correlated in each rice variety (*R*^2^-values was 0.73 and 0.68, all *P-*values < 0.01; Supplementary Figure S1). The results show that under heat stress conditions, the transcriptomic changes are relatively consistent within treatments (T36 vs T38). In order to validate the RNA-seq data and confirm the differential expression genes, we performed RT-qPCR on ten random candidate DEGs. The RT-qPCR results indicated that these candidate DEGs displayed the same expression patterns compared with the sequencing data, thereby confirming the reliability of the RNA-seq data (Supplementary Figure S2).

### The Importance of Maintaining the Homeostasis Balance of Metabolic Processes in Rice Resistance to Heat Damage

**FIGURE 4:**
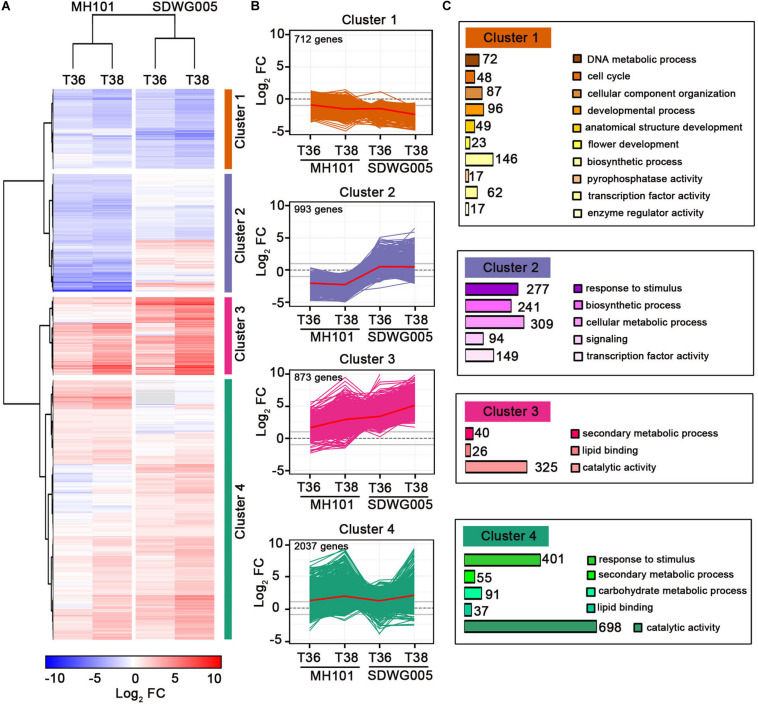
Global patterns of gene expression during heat stress. **(A)** Hierarchical clustering analysis to reveal the transcript expression patterns of DEGs in MH101 and SDWG005 spikelets subjected to different high temperature treatments (T36 or T38). Colored bars show the fold changes of gene expression under T36 and T38 treatments compared to their corresponding controls. **(B)** Gene expression profiles in clusters 1, 2, 3, and 4 as indicated. Lines reflected expression changes for each DEGs across different treatments. The mean changes of expression in each cluster are shown with a red line. **(C)** GO categories of DEGs enriched in each cluster. The number show the DEGs enriched in each GO categories.

To further characterize gene expression changes upon heat stress treatments and among rice varieties, we performed a hierarchical clustering by using the expression changes (log_2_FC value) for the identified 4615 DEGs shown as red number in Figure 3D (Supplementary Table S3). As shown in Figure 4A, analogous clusters with similar patterns of expression changes were readily observed. Therefore, we identified four clusters of DEGs, along with a red line reflecting the mean changes of expression describing each transcript accumulation pattern (Figure 4B). Cluster 1 included 712 DEGs that were progressively down-regulated under the two treatments (T36 and T38) in both genotypes, and the expression changes of these DEGs in SDWG005 were relatively more repressed than in MH101. Clusters 3 and 4 containing 873 and 2,037 DEGs, respectively, possessed most of the DEGs, and most of the DEGs were up-regulated by heat stress. The difference was that the genes in cluster 3 showed relatively higher induced expression levels in SDWG005 than in MH101 in response to heat stress. Interestingly, cluster 2 contained 993 DEGs, whose expression patterns under heat stress were contrary to that of SDWG005 and MH101, and the expression levels of SDWG005 were relatively higher than that of MH101 (Figure 4B).

To relate DEGs in clusters to biological functions, we performed a Gene Ontology (GO) enrichment analysis for biological process by agriGO (Du et al., 2010). The relative expression of DEGs in clusters 3 and 4, which mainly participated in secondary metabolic processes, lipid binding, and catalytic activity, were up-regulated in response to heat stress (Figure 4C). The DEGs belonging to stimuli responses and carbohydrate metabolic processes were also greatly enriched in cluster 4. The enriched GO terms of cluster 1 indicated that some genes involved in metabolic processes (catabolism and biosynthesis), transcription factor activity, cellular component organization, and developmental growth were repressed upon heat stress in both genotypes. By comparison, in cluster 2, enriched GO terms involved in processes included response to stimulus, biosynthetic process, cellular metabolic process, signaling, and transcription activity (Figure 4C). Interestingly, the relative expression value were higher in heat-tolerant SDWG005 than in heat-susceptible MH101 The results indicated that compared with MH101, heat-tolerant rice SDWG005 might still be able to establish a new steady-state balance of metabolic processes under heat stress condition.

### Identification of the Common and Genotype-Specific Mechanisms That Associated With Resistance to Heat

**FIGURE 5:**
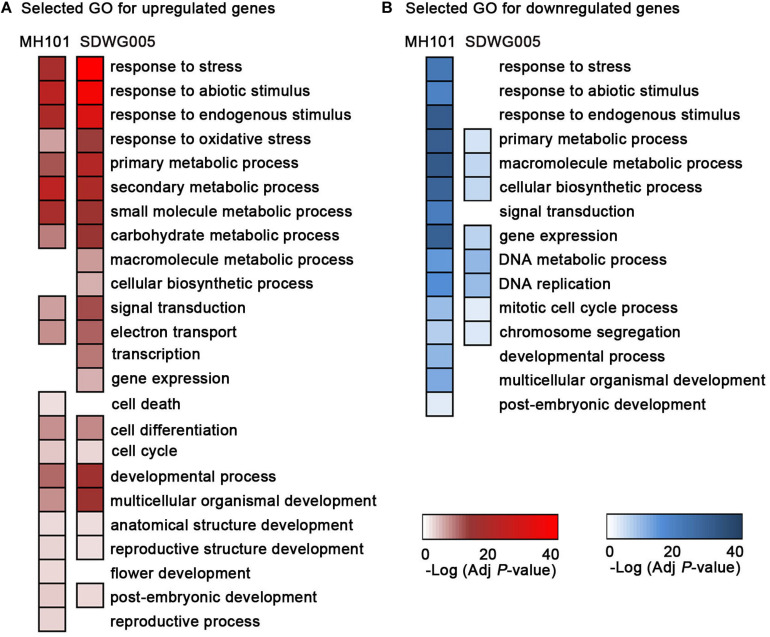
GO enrichment analysis of DEGs for the biological process using AgriGO. Most representative enriched GO categories from the up-regulated DEGs **(A)** or down-regulated DEGs in rice spikelets upon heat stress **(B)**, respectively. Color bars: -log_10_ transformed *P*-value (adjusted by Benjamini-Hochberg).

To further investigate the biological pathways of the up- or down-regulated DEGs in heat-tolerant and heat-sensitive varieties (Supplementary Table S4), we performed the GO and KEGG enrichment analyses. The representative GO categories enriched in SDWG005 and MH101 were selected respectively, and the heatmaps representing the log_10_-transformed *P*-values were constructed (Figure 5). We found that among up-regulated DEGs, GO terms associated with response to abiotic stress, oxidative stress, multiple metabolic processes, signal transduction, electron transport, and reproductive related development processes were more significantly enriched in tolerant variety SDWG005 (Figure 5A). While the GO terms involved in cell death, flower development, and reproductive process were specifically enriched in susceptible rice MH101 in response to high temperature (Figure 5A). On the other hand, GO terms for the down-regulated DEGs correlated with response to abiotic stress, signal transduction and reproductive development processes were also particularly observed in MH101 (Figure 5B). And GO terms for the down-regulated DEGs, including multiple cellular metabolic processes, DNA metabolic process, DNA replication, mitotic cell cycle process, and chromosome segregation, were more significantly enriched in MH101 than in SDWG005 (Figure 5B).

**FIGURE 6:**
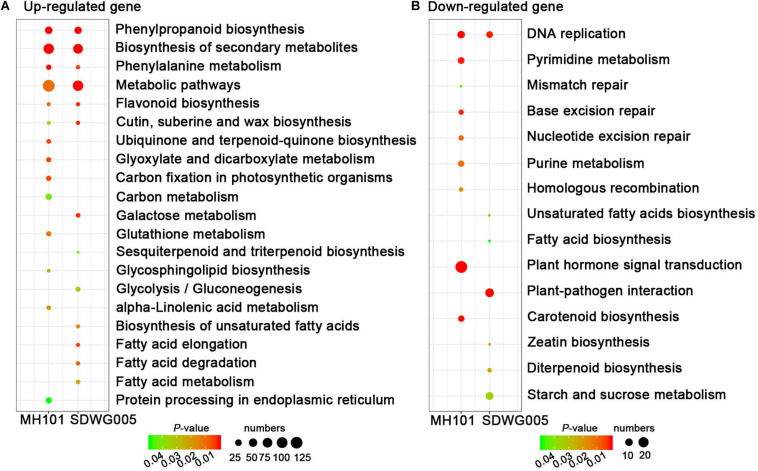
The KEGG pathway enrichment scatter plots of DEGs. KEGG pathway enrichment analyses for up-regulated **(A)** or down-regulated genes **(B)**, respectively. Significance is indicated by color, and the number of DEGs is indicated by the size of a circle.

We further determined whether the heat-responsive DEGs were involved in specific pathways by searching against the KEGG pathway database. The results showed that among the up-regulated DEGs, six significant KEGG pathways (FDR adjusted *P*-value ≤ 5%), including phenylpropanoid biosynthesis, phenylalanine metabolism, flavonoid biosynthesis, metabolic pathways, biosynthesis of secondary metabolites, and cutin, suberin and wax biosynthesis, were collectively enriched in both genotypes, indicating the common mechanisms exist in both tolerant and sensitive varieties that were associated with resistance to heat stress (Figure 6A and Supplementary Table S5). While only one pathway of DNA replication was collectively enriched in both two genotypes among down-regulated DEGs (Figure 6B and Supplementary Table S5). Notably, the pathways involved in DNA repair processes, including mismatch repair, base excision repair, nucleotide excision repair, and pyrimidine and purine metabolism, were exclusively enriched in sensitive cultivar MH101, indicating that heat-induced abnormal anther development and deformed pollen grains formation, possibly act through suppressing the DNA replication process and even disturbing DNA repair proofreading to harm genome integrity (Figure 6B and Supplementary Table S5).

Taken together, these findings suggested both the common and genotype-specific mechanisms existed in both tolerant and sensitive varieties that were associated with resistance to heat stress. In tolerant cultivar SDWG005, the resistance mechanisms were associated with reprogramming the cellular activities, such as response to abiotic stress, metabolic reorganization, and secondary metabolites biosynthesis. In contrast, heat responses in susceptible cultivar MH101 were mainly associated with reproductive development injury, which appeared to act through inhibiting the transcriptional levels of the genes involved in DNA replication and DNA repair proofreading, to impair the processes of post-embryonic and reproductive structure development.

### Analysis of DEGs Involved in Lignin and Flavonoids Biosynthetic Pathways

**FIGURE 7:**
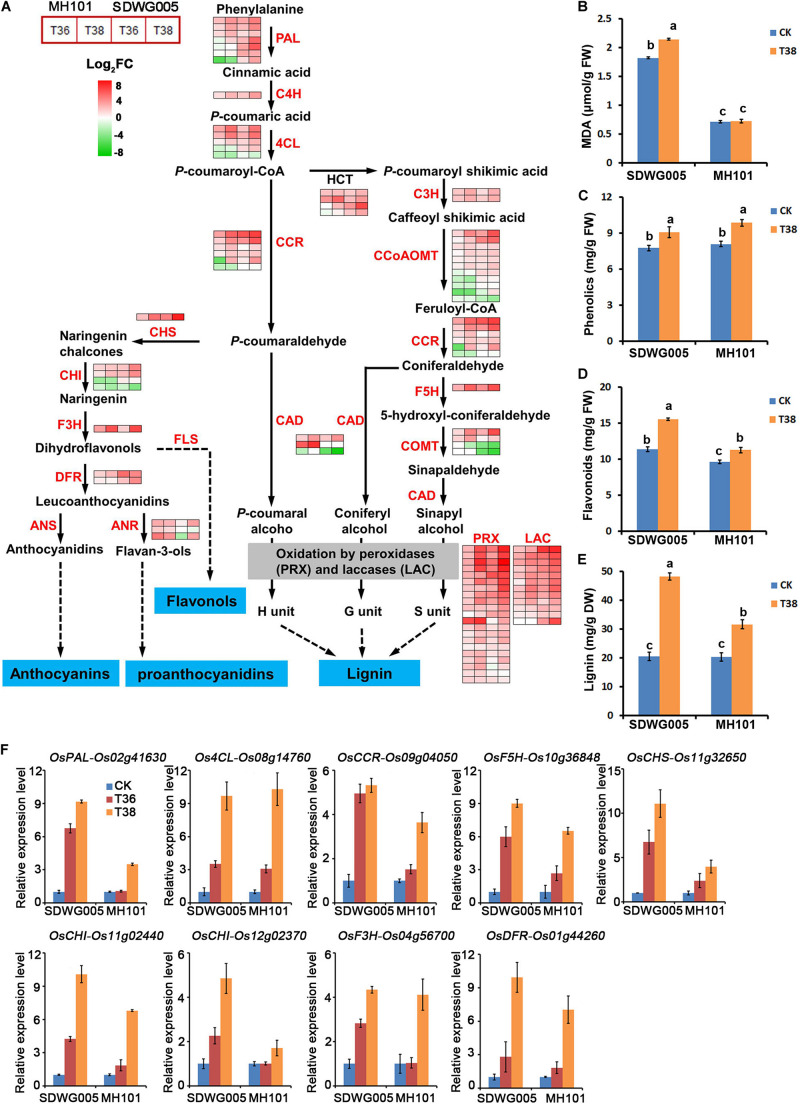
Transcriptional changes of DEGs involved lignin and flavonoids biosynthetic pathways. **(A)** Expression heatmap of representative genes related to the lignin and flavonoids biosynthetic pathways. Data were log_2_ transformed and normalized to those of the corresponding normal growth controls to determine fold changes, as indicated by the colored bar. The detailed expression patterns and identities of the genes in each of these biosynthetic pathways are shown in Supplementary Table S6. Total content of MDA **(B)**, phenolics **(C)**, flavonoids **(D)**, and lignin **(E)** in spikelets of SDWG005 and MH101 varieties under heat treatment for three days. **(F)** The relative expression levels of *OsPAL*, *Os4CL*, *OsCCR*, *OsF5H*, *OsCHS*, *OsCHI*, *OsF3H*, and *OsDFR* in spikelets of SDWG005 and MH101 were determined by qRT-PCR under T36 or T38 conditions for three days. The *O. sativa* β-tubline gene was used for normalization. Each value represents the mean of three independent observations and SD determined. Significant differences were calculated by One-way ANOVA with Duncan’s multiple test (SAS Institute, Inc., Cary, NC, United States). Different letters indicate the statistical difference between samples at *P* = 0.05.

To further investigate the key genes involved in thermotolerance and their regulation pathway, the genes involved in the most significant enriched phenylpropanoid biosynthesis pathway were analyzed. The DEGs involved in phenylpropanoid biosynthesis were screened, and identified 77 DEGs that included almost all the key enzymes related to lignin biosynthesis (Supplementary Table S6). In response to heat stress, a dramatic up-regulation of most of these unigenes was observed in both genotypes, especially under T38 treatment (Figure 7A). Almost all of the laccase (LAC, 12 unigene) and peroxidase (PRX, 21 unigenes) family members exhibited a significantly heat-inducible response. Moreover, 11 DEGs were identified involving in flavonoid biosynthesis pathway, another branch of phenylpropane biosynthesis pathway, which was also enriched in KEGG pathway analysis (Figure 7A and Supplementary Table S6). Except for two chalcone isomerase (CHI) unigenes, 9 of 11 the unigenes were up-regulated upon heat stress, especially for the CHS and F3H unigenes, which exhibited a higher heat-inducible expression under T38 treatment (Figure 7A). Whether in lignin biosynthesis or flavonoids biosynthetic, more up-regulated genes were observed in SDWG005 than MH101 upon heat stress.

The DEGs related to the six main heat-responsive TFs were analyzed, such as MYBs, NACs, WRKYs, and bHLHs families, which might be involved in the regulation of flavonoid and lignin biosynthesis pathways (Xu et al., 2015; Yoon et al., 2015; Wessels et al., 2019). A total of 52 MYBs, 49 NACs, 21 WRKYs, 51 ERFs, 12 HSFs, and 39 bHLHs TFs were identified (Supplementary Table S7). Results indicated that except for partial bHLH TFs gene family members, the majority gene family members of MYBs, NACs, WRKYs, and ERFs showed significantly regulated under heat stresses (Supplementary Figure S3). HSFs encoding heat shock proteins, as direct transcriptional activators of genes regulated by thermal stress, whose expression levels showed higher up-regulated in SDWG005 than in MH101 (Supplementary Figure S3). The different expression levels of these TFs genes, especially participated in the regulation of flavonoid and lignin biosynthesis pathways, may be one factor that led to their different thermotolerance in the two genotypes during pollen development upon heat stress.

We then further analyzed the content of oxidative stress marker MDA, anti-oxidants (total phenolics and total flavonoids) and lignin in spikelets under heat stress. We found that lipid peroxidation (MDA concentration) of SDWG005 was always higher than that of MH101 whether under heat stress treatment or not (Figure 7B). Although the concentration of total phenolics was significantly accumulated under T38 treatment, no significant difference was observed between SDWG005 and MH101 (Figure 7C). While a higher accumulation of total flavonoids were observed in SDWG005 under normal growth conditions and heat stress (Figure 7D). Moreover, a more lignin deposition happened in SDWG005 than MH101 under T38 treatment (Figure 7E). The transcriptional profile of nine genes (*OsPAL*, *Os4CL*, *OsCCR*, *OsF5H*, *OsCHS*, *OsCHI*, *OsF3H*, and *OsDFR*) involved in lignin and flavonoid biosynthesis pathway were further confirmed by using RT-qPCR. As illustrated in Figure 7F, a dramatic upregulation of all these genes in two genotypes was observed in response to high temperature, especially in T38 treatment. Except for *Os4CL* (*Os08g14760*), the induction response of the remaining 8 genes in SDWG005 was higher than in MH101. Collectively, the genotype-dependent difference in biosynthesis and accumulation of lignin and flavonoids might be vital factors that led to their different thermotolerance in the two genotypes, suggesting the lignin and flavonoid biosynthetic pathways might play important roles in rice heat resistance during reproductive stage.

## Discussion

With global warming growing more severe, heat stress has profound effects on crop growth and development, even leads to significant yield loss worldwide (Fahad et al., 2017; Wang and Qin, 2017). Despite great advances on physiological and biochemical aspects of the high temperature induced fertility injury in rice, there is limited information regarding the molecular mechanism responding to heat stress (Satake and Yoshida, 1978; Matsui et al., 2000; Jagadish et al., 2010; Yu et al., 2016; Yan et al., 2017). Based on previous reports, SDWG005 and MH101 showed contrasting thermotolerance differences during the flowering stage (Zha et al., 2016). Recently, a transcriptional profile analysis under different time courses of heat treatment on the anthers of SDWG005 at anthesis were conducted and identified agmatine-coumarin-acyltransferase gene *OsACT* may involve in SDWG005 thermotolerance (Liu et al., 2020). Here, we further characterized the physiological observation and transcriptomic expression patterns analysis on spikelets of SDWG005 during meiosis under different high-temperature treatment. We observed higher pollen viability in SDWG005 after heat stress at meiosis stage than in sensitive cultivar MH101 (Figure 1). The pollination properties analysis also showed that SDWG005 was much more tolerant to heat stress than MH101. Through comparison among treatments, the number of DEGs was higher in SDWG005 than in MH101 (Figure 3), which might indicate that heat-tolerant rice SDWG005 had a more complex response to heat stress than the susceptible rice MH101. Unlike the *aus indica* type rice variety “Nagina22” (“N22”) (Bahuguna et al., 2015), SDWG005 was highly heat tolerant but did not come with obvious poor agronomic performances. Hence, identification of key mechanisms of heat stress response in SDWG005 and understanding the molecular basis in such a novel extremely strong heat-resistant rice germplasm will help us to bring its thermotolerance into existing rice breeding programs.

It was reported that reproductive organs undergone both general and specific transcriptional alterations in response to high-temperature changes (Alba et al., 2004; Oshino et al., 2007; Endo et al., 2009; Bita et al., 2011). The general transcriptional changes refer to reprogramming of cellular activities by the activation of a number of genes related to stress perception, downstream signaling transductional and transcriptional controls, while the specific transcriptional changes involve silencing of cell proliferation- and DNA replication-regulatory genes (Abiko et al., 2005; Oshino et al., 2007; Zhang et al., 2012). Our results indicated that both common and genotype-specific mechanisms associated with resistance to heat stress existed in both tolerant and sensitive varieties. In the hierarchical clustering analysis, we observed that many DEGs, related to response to stimulus, biosynthetic process, cellular metabolic process, signaling, and transcription activity, showed the opposite pattern of expression (Figure 4), which indicated that heat-tolerant rice SDWG005 possibly established a new steady-state balance of metabolic processes to protect and repair proteins and membranes.

The GO and KEGG analyses further indicated that the up-regulated DEGs involved in multiple metabolic processes, transcription, and gene expression had higher expression in tolerant variety, which suggested that these events of rapid reprogramming of cellular activities possibly performed important roles in conferring thermotolerance (Figures 5, 6). Whereas the enriched down-regulated DEGs involved in DNA replication, mitotic cell cycle process, chromosome segregation, and DNA repair protection mechanism were significantly inhibited in sensitive rice plants (Figures 5, 6). It has reported that the genes that were down-regulated in response to high temperatures were predominantly expressed in tapetal cells, and the tapetum function might be impaired during microsporogenesis under high temperature (Endo et al., 2009). To determine whether the genes in the tapetum were affected under our high temperatures conditions, three known tapetum-specific genes, such as *TIP2*, *TDR* and *MTR*, were chose to examine the expression after exposure to high temperatures by RT-qPCR analysis. *TDR* and *TIP2* play a central role in differentiation, morphogenesis, and degradation of anther tapetum degradation (Li et al., 2006; Fu et al., 2014), and *MTR1* is thought to be involved in controlling the development of sporophytic and reproductive cells in rice (Tan et al., 2012). Compared with the normal growth control, the expression levels of *TDR* and *MTR1* showed slightly induction under T36 heat stress and recovered back to normal under T38 heat stress in SDWG005, while displayed abnormal high temperature-repressed expression patterns in MH101 (Supplementary Figure S4). A clear inhibition of *TIP2* was observed in both varieties under T38 heat stress, even though this gene was shown to be slightly induced in SDWG005 under T36 (Supplementary Figure S4). This was consistent with what we found previously that the flower development and reproductive process were impaired in susceptible rice MH101 in response to high temperature through GO and KEGG analyses, which indicated that the genotype-specific mechanisms might be responsible for the heat stress-induced pollen abortion and spikelets sterility.

The phenylpropanoid pathway is indispensable to plants, because of its role in the biosynthesis of lignin and the production of many other important compounds, such as the flavonoids, coumarins, and lignans (Vogt, 2010). The pathway begins with three reactions leading to the synthesis of *p*-coumaroyl CoA, the direct precursor for flavonoid or H-lignin biosynthesis, which represents the most important branch point within the central phenylpropanoid biosynthesis in plant. In this study, the phenylpropanoid biosynthesis pathway was the most significantly enriched pathway (Figure 6A). We further identified 77 DEGs incorporated all the key enzymes of lignin biosynthesis, and 11 DEGs related to flavonoids biosynthesis genes (Figure 7A). Some studies have demonstrated that cell wall remodeling proteins with enzymatic activity were involved in the modification of cell wall components in response to heat stress, which induced heat shock proteins expression and cell-wall remodeling to retain plasma membrane integrity, thus preventing cellular content leakage and conferring thermoprotection (Wu and Jinn, 2010; Wu et al., 2010; Huang et al., 2017). Here, we found that the expression levels of these genes related to lignin biosynthesis were highly induced in SDWG005, and more lignin were deposited in SDWG005 under heat stress (Figures 7A,E,F). The results were consistent with the notion that cell wall remodeling had a pronounced effect on thermotolerance, and will provide a foundation for further researches on fine-tuning the regulatory mechanisms to adjust the accumulation of lignin in response to heat stress.

Flavonoids are a class of secondary metabolites that are common in the plant kingdom, which as antioxidants can regulate the ROS levels and early anther development (Thompson et al., 2010; Ning et al., 2018). ROS are described as both signaling molecules and molecules that cause damage to cellular components, and it is generally well-accepted that correctly timed accumulation of ROS is beneficial for tapetum degradation and pollen viability (Satake and Yoshida, 1978; Saini et al., 1984; Zhao et al., 2018). In comparison to MH101, the decline of heat-induced injury of ROS scavenging enzymatic activity was not clearly observed in SDWG005 (Figure 2), indicating the ROS scavenging capacity in SDWG005 was better maintained for protecting the sensitive reproductive organs from heat-induced damage under heat stress. Moreover, more flavonoids were accumulated in SDWG005 under heat stress, indicating that the increased level of flavonoids related to the heat thermotolerance in rice (Figure 7D). Hence, we deduced that SDWG005 has a stronger defense ability to scavenge ROS molecules efficiently than MH101. The possible reason for the elevated heat tolerance of SDWG005 could be explained by the combination of two factors: Firstly, the increase of flavonoids biosynthesis and its accumulation in SDWG005 might contribute greatly to the enhancements of heat tolerance under heat stress, because of flavonoids being widely considered as non-enzymatic antioxidant molecules in plant cells; Secondly, the increase of flavonoids biosynthesis in SDWG005 may also play a regulatory role in the activation of the antioxidant enzyme system, because the antioxidant enzymes (SOD, CAT and APX) had been widely considered as an important mechanism actively used by plants to detoxify ROS damage. Several previous studies had also reported that the accumulation of flavonoids was closely associated with the activities of SOD and CAT for better antioxidant defense system under stressful environment (Winkel-Shirley, 2002; Dixon et al., 2005; Agati et al., 2011; Zaidi et al., 2019). Thus, the more accumulation of flavonoids in spikelets of SDWG005 may also stimulate the activation of the antioxidant enzyme system, and this occurrence was another important reason for the elevated heat tolerance of SDWG005 under the high temperature exposure at reproductive stage.

## Conclusion

Overall, heat-responsive transcriptome analysis of the spikelets of SDWG005, a novel extremely strong heat-resistant rice germplasm, illustrated the molecular mechanism of its thermotolerance. Genes involved in regulation of reprogramming the cellular activities, such as response to abiotic stress, metabolic reorganization, and secondary metabolites biosynthesis, were significantly up-regulated in thermotolerant variety with heat treatment. In contrast, in sensitive variety, the down-regulation genes associated with DNA replication and DNA repair proofreading could cause serious injury to reproductive development when exposed to high temperature during meiosis. Additionally, we found that the lignin and flavonoids levels played an important role in heat-induced defense responses. The results will provide useful information for the research on the mechanism of heat resistance and the application of heat resistance genes in rice breeding.

## Data Availability Statement

The datasets presented in this study can be found in online repositories. The names of the repository/repositories and accession number(s) can be found below: https://www.ncbi.nlm.nih.gov/, PRJNA633211.

## Author Contributions

SD and ZC designed and performed the experiments, analyzed the data, and prepared figures and tables. ZC performed the phenotyping experiments. TL, XF, and FH assisted in the phenotyping experiments. SD, XT, and HW wrote the manuscript. All authors read and approved the final manuscript.

## Conflict of Interest

The authors declare that the research was conducted in the absence of any commercial or financial relationships that could be construed as a potential conflict of interest.

## Footnotes

1 http://rice.plantbiology.msu.edu
