# Supplementary material for: Transcriptomic Analysis Reveals Important Roles of Lignin and Flavonoid Biosynthetic Pathways in Rice Thermotolerance During Reproductive Stage
Source: Front Genet. 2020 Sep 15;11:562937. doi: 10.3389/fgene.2020.562937 (PMC7522568; doi:10.3389/fgene.2020.562937)
Supplement: Supplementary file 1 [file Data_Sheet_1.PDF]

Supplementary Material

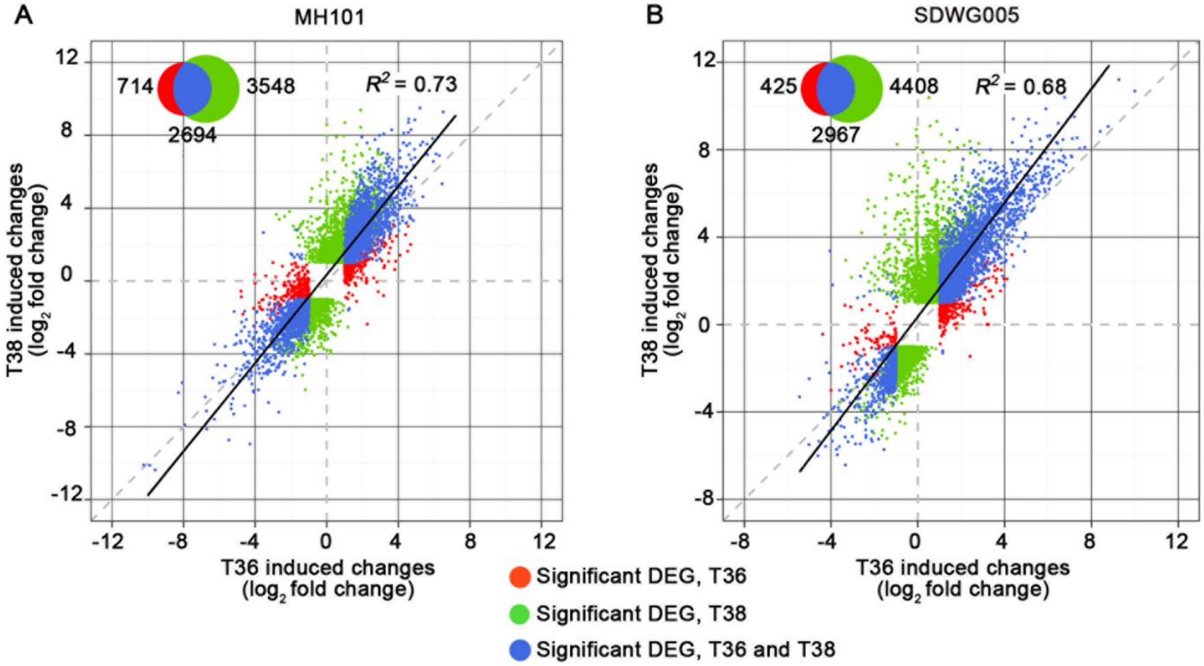

Figure S1. Comparison of the changes in gene expression induced by T36 and T38 treatments. Scatter plots of DEGs with significant changes (log_2FC values) show the comparison of T36 vs. CK and T38 vs. CK for MH101 (A) and SDWG005 (B) in response to HS.

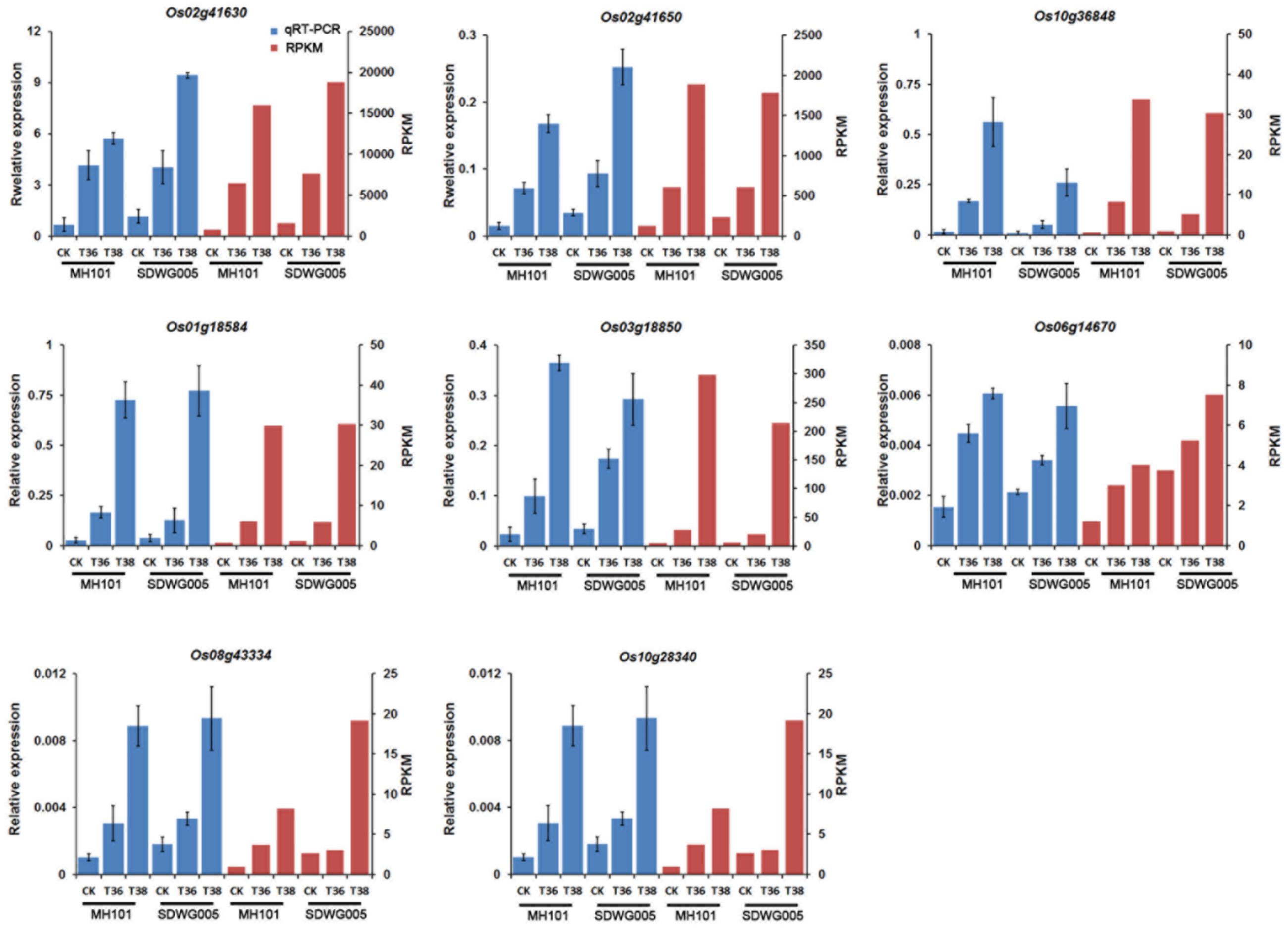

Figure S2. qRT-PCR and RNA-seq comparison.

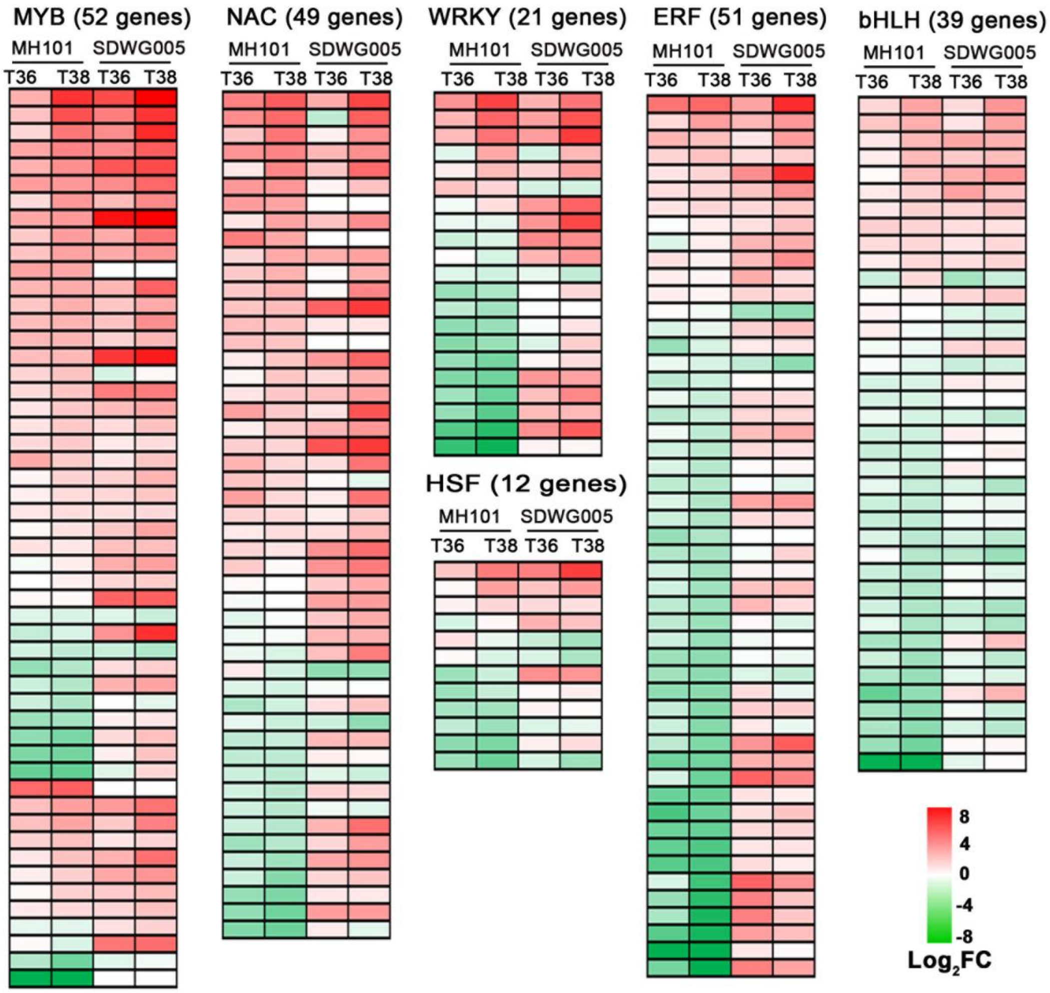

Figure S3. Heatmap representing transcriptional changes in the DEGs related to the six main transcription factor (TFs), including MYBs, NACs, WRKYs, HSFs, ERFs, bHLHs. Data were log transformed and normalized to those of the corresponding normal growth controls to determine fold changes.

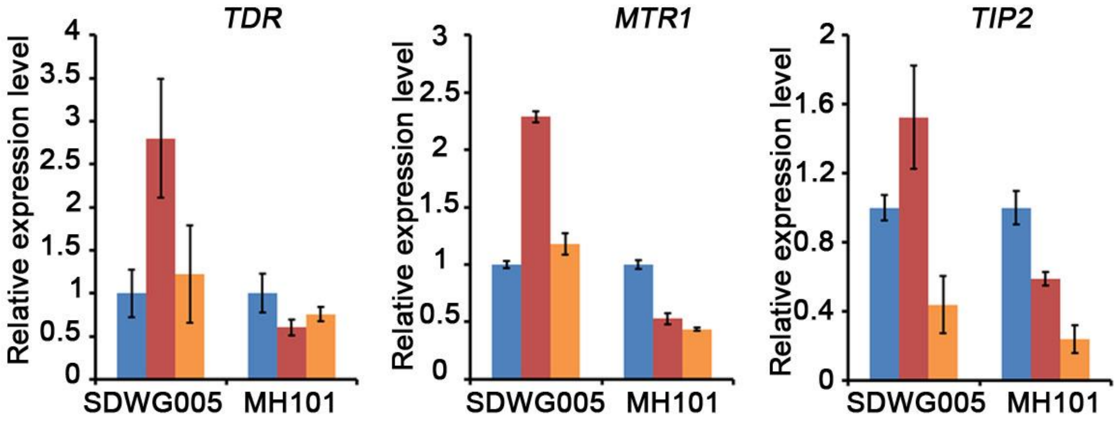

Figure S4. The relative expression levels of *TDR*, *MTR1*, and *TIP2* in spikelets of SDWG005 and MH101 under T36 or T38 conditions for three days were determined by qRT-PCR.
